# A paradigmatic autistic phenotype associated with loss of *PCDH11Y* and *NLGN4Y* genes

**DOI:** 10.1186/s12920-021-00934-x

**Published:** 2021-04-08

**Authors:** Rosaria Nardello, Vincenzo Antona, Giuseppe Donato Mangano, Vincenzo Salpietro, Salvatore Mangano, Antonina Fontana

**Affiliations:** 1 Child Neuropsychiatry Unit, Department Pro.M.I.S.E. “G. D’Alessandro”, University of Palermo, Palermo, Italy; 2 Department of Molecular Neuroscience, UCL Institute of Neurology, London, UK

**Keywords:** Autism spectrum disorder, Protocadherin, Neuroligin, Developmental global delay, Mixed gonadal dysgenesis

## Abstract

**Background:**

Most studies relative to Y chromosome abnormalities are focused on the sexual developmental disorders. Recently, a few studies suggest that some genes located on Y chromosome may be related to different neurodevelopment disorders.

**Case presentation:**

We report a child with sexual developmental disorder associated with a peculiar phenotype characterized by severe language impairment and autistic behaviour associated with a mosaicism [45,X(11)/46,XY(89)] and a partial deletion of the short and long arm of Y chromosome (del Yp11.31q11.23) that also involves the loss of both *PCDH11Y* and *NLGN4Y* genes. To our knowledge no study has ever reported the occurrence of the lack of both *PCDH11Y* and *NLGN4Y* located in the Y chromosome in the same patient.

**Conclusions:**

We hypothesized a functional complementary role of *PCDH11Y* and *NLGN4Y* within formation/maturation of the cerebral cortex. The impairment of early language development may be mainly related to the lack of *PCDH11Y* that underlies the early language network development and the later appearance of the autistic behaviour may be mainly related to deficit of inhibitory glicinergic neurotransmission *NLGN4Y-*linked.

**Supplementary Information:**

The online version contains supplementary material available at 10.1186/s12920-021-00934-x.

## Background

Mixed gonadal dysgenesis 45,X/46,XY is a rare development sex disorder (DSD) resulting from Y chromosome mosaicism, which induces abnormal gonadal development characterized by a variable phenotype ranging from partial virilization and ambiguous genitalia to freely male or female phenotypes [1]. Although there is a clear physiological and behavioral difference between men and women, the studies regarding the cerebral dysfunctions mediated by the Y-linked genes have elicited less interest than those mediated by the X-linked genes which are more numerous and along known as related to mental functioning [2].

Recently, the role played by the Y chromosome in sexual development has been recognized in brain development too; the two X–Y homologous gene pair, *PCDH11X/Y* and *NLGN4X/Y,* are emerging as two important families of genes, protocadherins and neuroligins, involved in neurodevelopmental disorders (schizophrenia, autism, intellectual disability, and epilepsy) although the clinical evidence is still rare [3]. Both genes code for two distinct synaptic cell adhesion molecules that connect preand postsynaptic sites through homo or heterophilic interactions of their extracellular domains, mediate trans-synaptic signaling cascades, and regulate multiple aspects of synapse development, function, and plasticity that are essential for the neurotransmission [4, 5]. The main expression of both genes *PCDH11Y* and *NLGN4Y* in the brain and in the early stage of the development suggested a link with the neurodevelopmental disorders that are more frequent in boys [6, 7]. As the clinical studies on the Y chromosome rearrangements are mainly directed towards the sexual development, here we present a child with mixed gonadal dysgenesis and a severe phenotype with developmental delay, language impairment, and autism spectrum disorder (ASD), recalling a regressive subtype, associated with a mosaicism [45,X(11)/46,XY(89)] and partial deletion of the short and long arm of Y chromosome (del Yp11.31q11.23). The concurrent loss of both *PCDH11Y* and *NLGN4Y* genes, involved in the formation/maturation of the brain, has never been described before, to our knowledge.

## Case presentation

The proband, a 30 months old child, is the only child born to healthy Italian unrelated parents. He was born at 40 weeks of gestation by normal vaginal delivery following a pregnancy complicated at the seventh month by two bleeding events. At birth the weight was 3950 g, the length was 50 cm, and the cranial circumference was 35.5 cm. The clinical postnatal course was uneventful. From 9 months of age the child was patient of our Department for a developmental delay: he acquired the head control at 4 months of age, but he was still unable to sit. On neuropsychiatric examination the patient revealed hypotonia and dysmorphic features including frontal bossing, triangular facies, large pinnas, right cryptorchidism and hypospadias. Abdominal ultrasound detected in the pelvic cavity a hypoechogenic areas of elongated shape consistent with a persistent Müllerian duct.

The echocardiogram revealed a patent foramen ovale. EEG showed a regular background activity and normal sleep patterns.

At 11 months the child was alert to his environment, interacted with the examiner and her mother through eye contact and vocalizations. He turned his head to sound, vocalized undifferentiated nasal sound and two different vowel sounds; he smiled when the examiner smiled and vocalized when the examiner spoke. He transferred objects from hand to hand, but could not grasp them. He could roll over and twist his trunk, he could sit just for 60 s. The child could not crawl and did not make early stepping movements. A formal development assessment by the Bayley Scales of Infant and Toddler Development—Third Edition showed performance in the average range at the cognitive scale (PC 100). His general performance on language scale was below average range (86 PC), but receptive language skills were appropriate to age, and expressive language skills were impaired (scaled score respectively 10 and 5). On motor scale the performance was extremely low (PC 61). At 25 months of age the child showed an erratic response to name and did not react to sudden loud noises. He showed inconsistent eye contact and displayed negative affect in response to either the test material and the examiner or caregiver. He avoided all physical contact, did not attempt to engage in social interaction, rarely vocalized nasal and vowel sounds, but without intentional communication. He showed stereotyped fingertip movements (rubbing), repetitive objects manipulation (stack of blocks), and tendency to focus attention on stimuli for too long. He could walk, but did not run or walk up/down stairs. He grasped pencil with his fingertips and scribbled. His performance on the Bayley III were deteriorated. Composite score on Cognitive and Language scales was 70 and 52 (below 2–3 sd) respectively. On Motor scale the performance was consistently extremely low (PC 64). Child’s ability to engage in self-regulation of emotions and behaviors, and child’s social competence was extremely low (Socioemotional scale PC 70). At 29 months a semi-structured standardized assessment of communication and social interaction (ADOS II toddler module) showed an overriding impairment in social-emotional reciprocity, no initiation of social interaction and no sharing of emotions, along with reduced or absent imitation of others' behavior, complete lack of speech, repetitive use of objects and reduced use of eye contact. The Comparison Score was 21 and indicated a moderate to severe level of risk for ASD. A conventional cytogenetic analysis, based on GTG-banded metaphases from peripheral blood lymphocytes of the patient at a 400-band resolution, revealed a mosaicism: 45,X(11)/46,XY(89) and a partial deletion of the short and long arm of Y chromosome (del Yp11.31q11.23). ArrayCGH was performed using Human Genome CGH Microarray Kit (8 × 60 K) (Agilent Technologies, Santa Clara, CA, USA), according to the manufacturer’s recommendations. The aCGH analysis, using Agilent Cyto Genomic Analytics software (V.3.0.6.6) showed a de novo partial deletion of 26 Mb in the chromosome Y from position 2,656,461 (Yp11.31) to 28,504,079 (Yq11.23) according to UCSC Genome Browser (hg19;GRCh Build 37, February 2009). The detailed data has been reported in “Additional file 1”.

## Discussion and conclusions

In the last decade, it is emerging that neurodevelopmental disorders, including ID and ASD, are mainly caused by genetic factors. The new techniques of genetic analysis, including chromosomal microarray and next-generation sequencing, have yield the identification of over 100 genes whose de novo postzygotic mutations, identified notably using the resequencing technologies, have significantly contributed to identifying the origins of many clinical features within the neurodevelopmental disorders [8]. A better understanding of the variants of uncertain significance (VUS), of the intronic variants, and of mosaic mutations will further contribute to the identifying of the genes responsible for the neurodevelopmental disorders [9].

Our patient with a karyotype 45,X/46,XY shows a clinical feature fulfilling the criteria of DSD identified as “congenital conditions in which development of chromosomal, gonadal, or anatomical sex is atypical” [1]. In addition, the proband has a gonadal dysgenesis, a well-known condition of an increased risk of malignant transformation; therefore, as also suggested by a recent investigation, he was at once referred within a specific surgical surveillance study [10]. In addition, he displays a severe phenotype characterized by language disorder, global developmental delay, poor social interaction, restricted interests, and repetitive and stereotyped behaviors with a peculiar clinical course that in some ways could evoke ASD subtype with regression of the main developmental functions. Most clinical studies regarding the 45,X/46,XY disorders, focused on the sexual development aspects. Recently, there is a growing evidence that some genes located on the Y chromosome are responsible for some neurodevelopmental disorders in addition to well-known DSD [3]. As our patient has an additional deletion of Y chromosome we tried to identify which role the lost genes could play in the cognitive and social development. We think that within the set of lacking genes the *PCDH11Y* and *NLGN4Y*, despite the incomplete knowledge of their molecular mechanisms, can play a significant role in the mental dysfunction of our patient.

Protocadherins (PCDH)11 are cell adhesion molecules belonging to the cadherin superfamily. Two types of PCDH11 were identified whose genes are located at Xq21.3 (*PCDH11X*) and at Yp11.2 (*PCDH11Y*) and share 98.1% nucleotide and 98.3% amino acids [11].

*PCDH11X/Y* mRNA is expressed in many regions of the fetal and adult human brain, including frontal, temporal, and occipital cortex, corpus callosum, amygdala, hippocampus, caudate nucleus, thalamus, substantia nigra, and cerebellum [4]. In addition, a longitudinal study of the prefrontal cortex using microarrays showed that levels of *PCDH11Y* change at different age, being highest in male neonates, and decrease through infancy and childhood [12]. The primary expression of *PCDH11X/Y* in the brain suggested a their significant role in the development and the functioning of the brain although many of their detailed molecular mechanisms are still unclear. Nevertheless, recent in vitro and in vivo studies documented that *PCDH11X* increased the neural proliferation, but decreased neural differentiation and premature migration, and that it exerted a negative regulator role of dendritic branching in cultured cortical neurons [13]. So, the *PCDH11X/Y* have been suggested as candidate genes for some neurodevelopmental disorders though the clinical accumulated data supporting this view are yet very rare [3].

Genetic analysis of fourteen developmental dyslexic patients disclosed that five individuals had a duplication (ranging 113–166 Kb) and one patient a deletion of 2 Mb at Xq21.3 including *PCDH11* gene [14].

In addition, it has been reported a male child with severe language delay, associated with a small deletion (Xq21.3 and Yp11.2) within the *PCDH11X/Y* gene. The X chromosome deletion was inherited from the unaffected carrier mother, but the Y chromosome deletion was a de novo occurrence [3].

This finding suggested that the *PCDH11X/Y* genes in humans play a role in language development and that rare copy number variation is a possible mechanism for communication disorders. Intriguingly, the deletion of Y chromosome of our patient extends to Yq11.23 region and includes the *NLGN4Y*, a family component of five postsynaptic cell-adhesion molecules (NL1, NL2, NL3, NL4 and NL4Y) that, interacting with the presynaptic neurexins, maintain trans-synaptic connection and function.

*NLGN4* and *NLGN4Y* transcripts were detected in all male brain regions without significant differences in regional distribution syndrome [15]. It has been reported that in mice the neuroligin-4 mRNA levels increase from embryonic to postnatal phase with a plateau at 3 weeks [7, 15].

Functional analyses showed that the loss of neuroligin-4 caused a profound and selective reduction in glycinergic synaptic transmission, interacting with inhibitory postsynaptic gephyrin and collybistin proteins, without impairing excitatory synaptic transmission [7]. A clinical screening for mutations in *NLGN4* in autistic individuals identified a frameshift *NLGN4X* mutation (1186insT) producing a stop codon at position 396 in two brothers of a Swedish family, one with typical autism and the other with Asperger syndrome [15]. Later, a 2-bp deletion in *NLGN4X* (1253delAG), leading to a premature stop codon encoding a truncating *NLGN4X* protein (D429X), was detected in 13 males from a French family with intellectual disability, including two males with autism and one with pervasive developmental disorder [16].

Interestingly, a *NLGN4Y* missense variant, (c.2035A > G p.I679V), without any variant in the homologous *NLGN4X* gene, was found in a patient with autism and in his father with learning disabilities [17].

Sadly, other studies did not disclose a high rate of *NLGN4* mutations in autistic patients. Hence, it is likely that *NLGN4* mutations may be responsible for a small subset of patients with autism, perhaps associated with a wider spectrum of neurodevelopmental disorders with mild to severe cognitive deficits.

The comparison of results of above mentioned studies suggests that the mutations of both *PCDH11Y* and *NLGN4Y* genes may be consistent with temporal patterns of onset of the clinical feature of our patient.

Indeed, our subject revealed an early and persistent impairment of language development suggesting a detrimental influence of *PCDH11Y* on the basic processes underlying the language maturation as above mentioned.

In addition, previous studies documented that the most inhibitory neurotransmission, mediated by GABAergic and glycinergic synapses, during the first 2–3 weeks of rodent postnatal development shifts from immature depolarizing to mature hyperpolarizing functioning and from GABAergic to glycinergic transmission [18]. This temporal pattern of late maturation of glycinergic neurotransmission seems to agree with the late appearance of autistic symptoms in our patient suggesting a possible relation between the lack of glycinergic inhibition *NLGN4-*modulated and the onset of autistic behavior. On the other hand, it has been reported that about one-third of ASD patients experienced a developmental regression between 15 and 24 months whose causal mechanism remains elusive although a genetic contribution including genes coding for post synaptic density proteins, is recently emerging with strength [19]. In conclusion, our data cautiously suggest that both genes may play a complementary functional role in the development of our patient's disorder.

## Supplementary Information

**Additional file 1:** Detailed information about genetic tests.

## Data Availability

The datasets analyzed during the current study are available from the corresponding author on reasonable request at Department Pro.M.I.S.E. ‘‘G. D’Alessandro-Child Neuropsychiatry Unit. The detailed data has been reported in “Additional file 1”.

## Abbreviations

ASD: Autism spectrum disorder
DSD: Disorders of sex development
*PCDH11Y*: Protocadherins-11Y
*NLGN4Y*: Neuroligin-4Y

## References

[1] Lee PA, Houk CP, Ahmed SF, Hughes IA (2006). Consensus statement on management of intersex disorders. Pediatrics.

[2] Kopsida E, Stergiakouli E, Lynn PM, Wilkinson LS, Davies W (2009). The role of Y chromosome in brain function. Open Neuroendocrinol J.

[3] Speevak MD, Farrell SA (2011). Non-syndromic language delay in a child with disruption in the protocadherin11X/Y gene pair. Am J Med Genet Part B.

[4] Yoshido K, Sugano S (1999). Identification of a novel protocadherin gene (PCDH11) on the human XY homology region in Xq21.3. Genomics.

[5] Jamain S, Quach H, Betancur C, Råstam M, Colineaux C, Gillberg IC (2003). Mutations of the X-linked genes encoding neuroligins NLGN3 and NLGN4 are associated with autism. Nat Genet.

[6] Kim SY, Chung HS, Sun W, Kim H (2007). Spatiotemporal expression pattern of non-clustered protocadherin family members in the developing rat brain. Neuroscience.

[7] Zhang B, Gokce O, Hale WD, Brose N, Südhof TC (2018). Autism-associated neuroligin-4 mutation selectively impairs glycinergic synaptic transmission in mouse brainstem synapses. J Exp Med.

[8] Lim ET, Uddin M, De Rubeis S, Chan Y, Kamumbu AS, Zhang X (2017). Rates, distribution and implications of postzygotic mosaic mutations in autism spectrum disorder. Nat Neurosci.

[9] Rahman MM, Uddin KF, Al Jezawi NK, Karuvantevida N, Akter H, Dity NJ (2019). Gonadal mosaicism of large terminal de novo duplication and deletion in siblings with variable intellectual disability phenotypes. Mol Genet Genom Med.

[10] Matsumoto F, Matsuyama S, Matsui F, Yazawa K, Matsuoka K (2020). Variation of gonadal dysgenesis and tumor risk in patients with 45, X/46. XY Mosaicism Urol.

[11] Blanco P, Sargent CA, Boucher CA, Mitchell M, Affara NA (2000). Conservation of PCDHX in mammals; expression of human X/Y genes predominantly in brain. Mamm Genome.

[12] Weickert CS, Elashoff M, Richards AB, Sinclair D, Bahn S, Paabo S (2009). Transcriptome analysis of male female differences in prefrontal cortical development. Mol Psychiatry.

[13] Wu C, Niu L, Yan Z, Wang C, Liu N, Dai Y (2015). Pcdh11x negatively regulates dendritic branching. J Mol Neurosci.

[14] Veerappa AM, Saldanha M, Padakannaya P, Ramachandra NB (2013). Genome-wide copy number scan identifies disruption of PCDH11X in developmental dyslexia. Am J Med Genet Part B.

[15] Jamain S, Radyushkin K, Hammerschmidt K, Granon S, Boretius S, Varoqueaux F (2008). Reduced social interaction and ultrasonic communication in a mouse model of monogenic heritable autism. Proc Natl Acad Sci U S A.

[16] Laumonnier F, Bonnet-Brilhault F, Gomot M, Blanc R, David A, Moizard MP (2004). XLinked mental retardation and autism are associated with a mutation in the NLGN4 gene, a member of the neuroligin family. Am J Hum Genet.

[17] Yan J, Feng J, Schroer R, Li W, Skinner C, Schwartz CE (2008). Analysis of the neuroligin 4Y gene in patients with autism. Psychiatr Genet.

[18] Gamlin CR, Yu WQ, Wong ROL, Hoon M (2018). Assembly and maintenance of GABAergic and Glycinergic circuits in the mammalian nervous system. Neural Dev.

[19] Goin-Kochel RP, Trinh S, Barber S, Bernier R (2017). Gene disrupting mutations associated with regression in autism spectrum disorder. J Autism Dev Disord.

