# Supplementary material for: A paradigmatic autistic phenotype associated with loss of PCDH11Y and NLGN4Y genes
Source: BMC Med Genomics. 2021 Apr 8;14:98. doi: 10.1186/s12920-021-00934-x (PMC8034074; doi:10.1186/s12920-021-00934-x)
Supplement: Supplementary file 1 — Additional file 1: Detailed information about genetic tests. [file 12920_2021_934_MOESM1_ESM.doc]

**Supplementary Material**

ArrayCGH was performed using Human Genome CGH Microarray Kit (8×60K) (Agilent Technologies, Santa Clara, CA, USA), according to the manufacturer’s recommendations. The aCGH analysis, using Agilent Cyto Genomic Analytics software (V.3.0.6.6) showed a *de novo* partial deletion of 26Mb in the chromosome Y from position 2,656,461 (Yp11.31) to 28,504,079 (Yq11.23) according to UCSC Genome Browser (hg19;GRCh Build 37, February 2009).

Raw data were generated using Agilent Feature extraction and analysed by Cytogenomics 4.0.3.12 software using ADM-2 algorithm (Agilent Technologies, Santa Clara, CA, USA). To improve the accurancy of the results, the Diploid Peak Centralization was applied. Aberrations was considered if at least four adjacent probes were involved and the Minimum Absolute Log Ratio (MAALR) was +/- 0,25. Other specific filters and analysis parameters: Design level, Feature level, Intra-Array replicates, GC Correction.
